# Exploring the Use of Robotics in Reconstructive and Plastic Surgery: A Comprehensive Perspective

**DOI:** 10.7759/cureus.96571

**Published:** 2025-11-11

**Authors:** Yousef AlKarboli, Naomi Abara, Ali Alotaibi, Amar Shamsah, Ahtziri Pagan, Pawan Acharya, Tarini Mudunuri, Bashir Imam, Ruthel S Rose, Gurusha Jangid, Christian Mazzocchi, Bestin Thomas, Ramsha Ali, Mohammed H Alsayed, Mohammed Waseem

**Affiliations:** 1 Basic Sciences, University of Lancashire, Preston, GBR; 2 General and Vascular Surgery, Royal Cornwall Hospitals NHS Trust, Truro, GBR; 3 General Surgery, Al Adan Hospital, Ministry of Health, Mubarak Al-Kabeer, KWT; 4 Surgery, University of Medicine and Health Sciences, Basseterre, KNA; 5 Surgery, Charing Cross Hospital, London, GBR; 6 General Surgery, Sri Ramachandra Institute of Higher Education and Research, Chennai, IND; 7 Pediatrics, University of Pittsburgh Medical Center, Coudersport, USA; 8 Internal Medicine, Kingston Public Hospital, Kingston, JAM; 9 General Surgery, Dr. Sampurnanand Medical College, Jodhpur, IND; 10 Medicine and Surgery, Universidad Central de Venezuela, Caracas, VEN; 11 Medicine and Surgery, Sheikh Shakhbout Medical City, Abu Dhabi, ARE; 12 Medicine and Surgery, Peoples University of Medical & Health Sciences for Women, Nawabshah, PAK; 13 Medicine and Surgery, Sudan International University, Khartoum, SDN; 14 Medicine, Sree Balaji Medical College and Hospital, Chennai, IND

**Keywords:** anesthetic outcomes, breast reconstruction, lymphovenous anastomosis, microsurgery, postoperative recovery, robotic-assisted surgery, surgical precision

## Abstract

Rapid advancements have revolutionized robot-assisted surgery (RAS) in the healthcare field, owing to substantial investments in the technology. The development of RAS progressed alongside advancements in microscopes and instruments, enabling microsurgeons to perform supermicrosurgical procedures in a more precise and efficient manner. This paper critically appraises the contemporary literature on RAS, exploring the risks and benefits of integrating and investing in emerging robotic technologies within plastic and reconstructive surgery, along with its limitations and challenges. This narrative review includes systematic reviews, case series, and prospective and retrospective reviews retrieved from PubMed published within the last 10 years, written in English, and conducted in live human subjects. Studies addressed applications of RAS in microsurgery, head and neck reconstruction, breast reconstruction, lymphovenous anastomosis, and aesthetic procedures. The review emphasized clinical outcomes, ergonomic impacts, patient satisfaction, safety, complication rates, and technological innovations. The current literature suggests that RAS is comparable, if not superior, to conventional techniques with respect to safety and effectiveness in specific reconstructive procedures. Reported benefits encompass shorter hospital stays, fewer complications, improved cosmetic outcomes, and enhanced surgeon dexterity. However, challenges persist, such as steep learning curves, high acquisition and maintenance costs, and a lack of tactile feedback, although these limitations are slowly being overcome through continued research and technological innovations. RAS is revolutionizing plastic and reconstructive surgery by enhancing surgical precision, ergonomics, and patient recovery. However, widespread adoption is constrained by substantial costs and the pace of technological evolution. Evidence has shown how it has become an essential component of modern surgical practice. Future research should prioritize high-quality comparative trials, cost-effectiveness analyses, and training programs to facilitate broader clinical integration.

## Introduction and background

With the aid and use of robotic assistance, achieving what was thought of as impossible is now within our reach. Over the last two decades, there has been increasing momentum in the advancement of robot-assisted minimally invasive and precision surgery. Although the concept was first proposed in 1967, clinical utilization of surgical robots was not documented until the late 1980s [1]. Early applications were reported in orthopedics, urology, neurosurgery, and otolaryngology, and its role is now well established in some of these surgical specialties [1].

A study reports that the mean operative time was shorter for manual procedures than for the robot-assisted group [2]. This observation raises a question: Does robot-assisted surgery (RAS) improve surgical accuracy, patient outcome, and recovery compared to traditional surgical methods? 

In plastics and reconstructive surgery, robotics assistance is still emerging with an expanding breadth of applicability in reconstruction [3,4]. This is evidenced by an expanding body of research exploring applications of robotics across the field of plastic surgery, including breast reconstruction, lymphatic surgery, abdominal wall surgery, microsurgery, and flap harvest. 

Microsurgery has continued to advance since the first robot-assisted microsurgery done by the da Vinci Surgical system in 2007 [5]. Recent dedicated microsurgical robots, like the Symani Surgical System and MUSA robot, appear promising, offering higher precision and overcoming the technical demand of super microsurgical micro-calibre vessel anastomosis [2,6].

In comparison to traditional methods, several studies report reduced donor site morbidity, shorter inpatient stays, and equivalent or improved aesthetic and functional outcomes [7,8]. The use of robotics during surgery enhances precision and motion scaling; however, these benefits may be offset by longer operative times and higher costs [4]. Overall, robotic surgery is typically less invasive, improves access, and offers greater dexterity than conventional open approaches [3]. 

However, clinical adoption is restrained by factors such as high capital expenditure, insufficient specialized instruments, bulky systems needing increased operating-room space, steep learning curves, and prolonged operating time [9]. Furthermore, there remains a paucity of holistic literature assessing robotic methods versus standard traditional surgical methods in broader clinical and reconstructive practice.

Incorporating robotic assistance into the surgical field has prevailed in decreasing postoperative complications and decreasing the duration of time to complete operations [2,8,9]. A 2023 study reported substantial reductions in complications and damages to tissues and neurovascular structures [9]. With the rapid increase in research regarding the use of new-age technology in surgery, the majority conclude that emerging technologies enhance surgical accuracy and facilitate faster, smoother recovery [1,5,8].

Studies suggest that the implementation of RAS increases accuracy and holds considerable promise for the field of plastic and reconstructive surgery. However, limitations still exist. Nonetheless, high capital and consumable costs, particularly for microsurgical instruments, restrict adoption across institutions [3]. In addition, these systems require intensive training, creating an interface with its utility not being fully taken advantage of or being optimized entirely by plastic surgeons. If it is used more, it can be refined to the needs and specifications of plastic surgeons. Thus, cost, limited instrumentation, and the associated learning curve constrain widespread use of RAS [9]. 

Furthermore, the growth of RAS in plastic and reconstructive surgery is paralleled by uncertainty regarding its applicability across all subspecialties. As the implementation of RAS is concentrated in selected areas in the field of plastics and reconstructive surgery, it gives a skewed picture of RAS implementation in the specialty, resulting in a lack of confidence from doctors, policy-makers, and institutions to adopt and financially invest in the technology. As a result, there needs to be more comprehensive data that includes outcomes from a wider range of subspecialties in plastic and reconstructive surgery [3,9-13].

This narrative review combines the current main evidence-based data on the clinical effectiveness of RAS in plastic and reconstructive surgery. We hypothesize that RAS enhances surgical accuracy, improves cosmetic and functional outcomes, and extends procedural capabilities compared to conventional methods. Additionally, it is expected to shorten hospital stays, lower donor site morbidity, and provide a less invasive approach. Moreover, for microsurgeons, it offers more ergonomic advantages during prolonged operations. 

The integration of RAS signifies the ongoing evolution of plastic surgery as a specialty. To clarify the efficacy, efficiency, and safety of RAS, this narrative review offers a timely collation of contemporary clinical data, including randomized controlled trials, systematic reviews, and large-scale retrospective analyses. This review also aims to provide practical insights for implementation to surgeons, researchers, and hospitals seeking to implement this technology by listing both the limitations and potentials of RAS. 

## Review

A literature search was conducted on PubMed using the following terms: (“robotic surgery”) AND (“plastic surgery” OR “reconstructive surgery”).

We considered studies published within the last 10 years that involved human adults undergoing robotic-assisted reconstructive procedures. Exclusion criteria comprised studies using cadavers, children, or animals, and studies written in languages other than English. The database search identified 467 studies, and after screening, 18 studies met the inclusion criteria and were included in this narrative review.

The following sections discuss clinical feasibility, outcomes, technological advances, limitations, and future directions of RAS in plastic and reconstructive surgery.

Comparison of robot-assisted surgery versus traditional surgery

Surgical techniques in plastic and reconstructive surgery continually evolve to deliver greater precision, safety, and aesthetic excellence. RAS, which was previously limited to specialties such as gynecology and urology, represents a glimpse into the future of plastic and reconstructive surgery and is now becoming a desirable tool in various specialties, surpassing conventional procedures in various disciplines, including plastic and reconstructive surgery. Although conventional techniques continue to be the gold standard in many settings, the expanding body of clinical research and trials is steadily revealing the benefits that RAS confers on both surgeons and patients.

In the essence of comparing RAS to conventional techniques, a prospective database by Struebing et al. [14] recorded 100 consecutive cases of RAS in microsurgery at a high-volume academic centre to assess its clinical application and efficacy. It has demonstrated successful outcomes with only 2.7% of RAS that led to complete flap losses, and only 3% of cases needed to be switched to conventional surgery. However, this lacked clarity compared to baseline flap loss with conventional methods. K. Tolksdorf et al. [15] bridged this gap in a prospective single-centre study of flap procedures in cranio-maxillofacial surgery that showed no difference in the revision surgery rate (6.6%) and flap loss rate (3.3%) in both groups. These outcomes offer supportive evidence that RAS is feasible and safe for a wide range of microsurgical applications.

Additional head-to-head studies reinforce these findings. Yusufov et al. [7] conducted a systematic review and meta-analysis comparing robot-assisted and conventional harvesting of deep inferior epigastric perforator (DIEP) and latissimus dorsi (LD) flaps in breast reconstruction. Their outcomes demonstrated that RAS was associated with longer operative duration but reduced hospital stay. Roy et al. [11] supported this, indicating a reduction in complications, positive patient-reported outcomes, and shorter incisional length. Therefore, although RAS typically prolongs intraoperative time, its postoperative advantages appear to outweigh this limitation.

In transoral and retroperitoneal procedures, robotic approaches have demonstrated clear benefits in anatomically complex regions. For example, robotic arms may reach very narrow areas with enhanced articulation and visualization. Systems such as the da Vinci and Symani provide improved access and range of motion, enabling surgeons to perform reconstructions that would be challenging or riskier using open surgeries [10,16]. Awad et al. [10] highlighted that RAS eased procedural complexity in areas like the head and neck, where conventional surgical access is restricted.

In addition, robotic systems have features that are specifically beneficial in microsurgical tasks, such as mitigating tremors and reducing movement. These capabilities provide a level of sub-millimeter precision unattainable with the human hand alone. Könneker et al. [16] emphasized this point in their prospective series on extremities reconstructions, which is more challenging with conventional instruments, where robotic aid facilitated precise vascular dissection and anastomosis in deep and confined surgical space.

Conventional microsurgery imposes a significant ergonomic burden on surgeons, who spend hours hunched over an operating microscope, compromising posture, endurance, and concentration. In that regard, RAS provides a substantial enhancement by enabling surgeons to work comfortably from a seated position at a console, utilizing hand and foot controls to maneuver precision tools, diminishing musculoskeletal strain and fatigue, and preventing chronic pain. Wee et al. identified enhanced ergonomics in robotic surgery relative to laparoscopy [17], and Pérez-Salazar et al. suggested improved posture and reduced muscle fatigue in robotic settings [18]. Monfared et al. conducted an objective ergonomic evaluation, such as surface-electromyography analysis, which indicated reduced physical demand in robotic cases [19].

Furthermore, robotic technology can level the field for less experienced surgeons. According to Awad et al. [10], RAS systems assist surgeons with little microsurgical experience in executing fine procedures with enhanced precision and confidence. The utilization of these procedures may improve access to sophisticated reconstructive surgery in resource-limited settings.

Overall, current evidence indicates that RAS provides safer and precise access, enhanced ergonomics, and outcomes that are equivalent to, if not superior to, conventional surgery for several reconstructive treatments. Nonetheless, despite the strengths of robotic systems, it is crucial to recognize the limitations, which will be discussed in this review.

Role of RAS in reducing hospital stay and complications and improving aesthetic outcomes

RAS has demonstrated effectiveness in reducing hospital stay while maintaining comparable safety outcomes in breast reconstruction [7]. Kim et al. conducted a systematic review and meta-analysis comparing robot-assisted flap harvesting with conventional methods in breast reconstruction, encompassing 370 RAS and 772 conventional reconstructions. The RAS group showed a statistically significant reduction in hospital stay compared to the conventional group, with no increase in postoperative complications such as seroma, re-operation, delayed healing, infection, or hematoma. There was no significant difference between the groups, suggesting that the safety of RAS is comparable to that of conventional surgery in breast reconstruction. Therefore, RAS appears to be an effective and safe approach for breast reconstruction surgery with reduced hospital stay [7].

Awad et al. extended these observations in a meta-analysis that screened more than 2,000 studies and synthesized data from 176 eligible reports spanning lymph-node dissection, DIEP, LD, and superficial inferior epigastric artery (SIEA) flaps, vaginoplasty, craniofacial reconstruction, abdominal-wall repair, and transoral surgery [10]. Overall, they observed reductions in hospital stay, complications, blood loss, and scarring. For instance, robotic nipple-sparing mastectomy reported reduced postoperative complications and high patient satisfaction, mirroring outcomes in abdominal-wall and transoral reconstructions. A key factor underlying these outcomes is the use of minimally invasive techniques with smaller incisions, which not only reduces surgical trauma but also contributes to improved aesthetic results [10].

Similarly, a systematic review by Roy et al. on robotic flap harvest in autologous breast reconstruction (a total of 84 DIEP and 160 LD flaps), robotic cases demonstrated fewer complications; higher patient-reported satisfaction with overall outcomes, symmetry, and and scar appearance; and superior BREAST-Q scores (a validated, patient-reported outcome measure assessing satisfaction and quality of life after breast surgery) relative to conventional surgery, reflecting greater overall satisfaction [11].

Surgical accuracy and precision

Robot-assisted platforms bring about unparalleled precision through tremor filtration, motion scaling, and high-definition three-dimensional visualization, enabling sub-millimeter control in the most complex surgical scenarios.

A study observing 16 upper extremity reconstructions done with the Symani Robotic System observed that the method was highly efficient without sacrificing any accuracy. Operators attributed this to the device’s tremor elimination and motion scaling with 7-degree-of-freedom instruments. The robot allowed for sub-millimeter precision in vascular anastomosis. On average, arterial end-to-end anastomosis took 31 minutes, arterial end-to-side took 33 minutes, and venous anastomoses took 20 minutes - only seven minutes longer than conventional benchmarks. Most importantly, there were no complete or partial flap losses in comparison to the conventional methods, which have a complete flap loss rate of 6% and a partial of 8%. This implies that the RAS could potentially be safer and more efficient [20].

Subsequently, the first-in-human free-flap reconstruction with Symani involved anastomoses of 0.8-mm vessels, demonstrating accuracy on an ultra-fine scale. All the vessels operated on showed immediate and sustained patency, and there was no flap loss or need for re-exploration. The authors concluded that RAS makes anastomoses much more reproducible by mitigating physiological tremors [21].

These results have been supported consistently across independent studies, ensuring the observations are reproducible. In addition to enhanced precision, ergonomic benefits and improved outcomes have also been noted. Furthermore, robotic systems give surgeons better training opportunities and foster standardized surgical techniques and consistent outcomes [22].

Preclinical trials

​Preclinical trials are the springboard for testing robotic systems; they help identify technical limitations, optimize device performance, and establish standardized procedures that guarantee reproducibility. Ultimately, preclinical validation lays a strong foundation for successful clinical adoption.

Artificial vessel trials testing novice, resident, and experienced microsurgeons showed that using the RAS led to fewer microsurgical errors, finer anastomosis outcomes, and higher quality work. Anastomosis Lapse Index (ALI) is an objective assessment of the quality of microvascular anastomoses used in simulated training. Lower ALI scores were observed across the board with RAS, meaning fewer errors in higher-quality anastomoses [23].

The scope of robotic surgery extends beyond the established da Vinci and Symani systems. A team of researchers has developed a three-fingered robotic system designed to manipulate breast tissue achieved a mean targeting accuracy of 1.16 mm in a dynamic breast model [24]. This study highlights the potential of robotic systems to augment surgical dexterity and gives us an understanding of the future of robotic surgery.

Robotics' impact on patients’ recovery time

Postoperative outcome regarding the impact of recovery is still new. Within the plastics and reconstructive surgery specialty, each procedure has its own influence when it comes to robotics and its outcome.

The use of robotics has shown improvement, for example, with postoperative pain, as well as hospital length of stay when doing a muscle flap harvest [3]. When exploring the benefits of postoperative recovery, we must keep in mind that each procedure within the field can have different conclusions. As continued use of robotics improves, so will the knowledge and skills in the field, allowing a positive experience for patients and an outcome [3]. Lymphatic reconstruction is the best represented area right now in the plastic and reconstruction field, where procedures such as lymphaticovenular anastomosis done with robotics can be achieved via outpatient [6], which in turn can significantly shorten recovery time. Robotics approach not only helps with visual aesthetic but also the patient’s recovery by influencing the dexterity of the surgeon and allowing smaller scars in each patient. For breast reconstruction, incorporating robotic technology for DIEP flaps allows the incisions to be more precise, therefore accelerating postoperative recovery [7].

During robotic-assisted anastomosis for anterolateral thigh free flap, some complications were reported, therefore altering the timing for recovery. One case needing revision after an arterial and venous thrombosis was identified. Following a thrombectomy and re-anastomosis, the length of hospital stay postoperatively was on average 15.5 ± 10.8 days [4]. Thus far, the majority of the time procedures done with robotics show a reduction of hospital stay, but other factors will alter its incorporation into the field, notably the monetary investment in the equipment [7].

Relevance to modern-day clinical practice: importance and implications of results

RAS has widened the breadth of feasible procedures in the field through improved access to complex anatomical regions via minimal access points. This portrays the emergence of a new dimension in modern clinical practice that was previously considered unattainable or only achievable with significant morbidity.

As highlighted throughout this narrative review, RAS has demonstrated clinical improvement in outcomes with downtrend patterns seen in complication rates, postoperative pain, blood loss, and inpatient stay [10].

**Figure 1:**
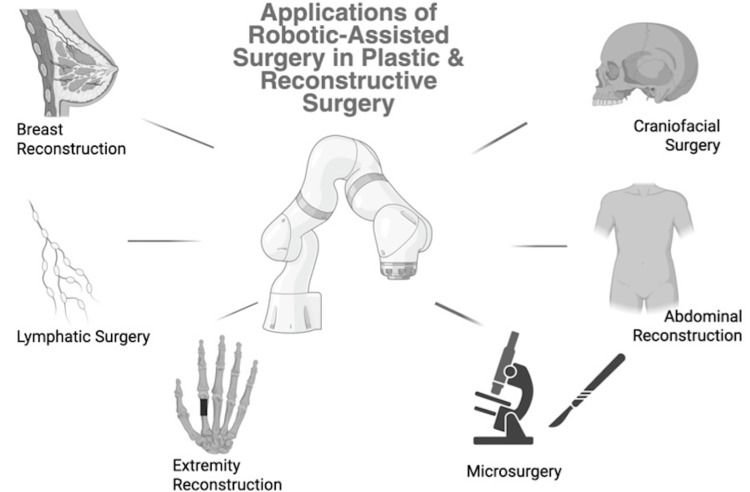
Applications of robotic-assisted surgery in plastic and reconstructive surgery Created in BioRender. Acharya P (2025). https://BioRender.com/1z5gvyq Publication License ID: ER28FJ2RGW

With this transformative impact of robotics-driven surgical solutions, it is easy to understand the growing and well-established role in current surgical practice. Furthermore, it accounts for the shift towards the increasing integration of robotic systems across certain subfields of plastic and reconstructive surgery to achieve these goals of modern surgical practice (Figure 1).

Head and neck reconstructive surgery

Prior to the advent of robotic assistance in cavity surgery, resection of nasopharyngeal and oropharyngeal tumors with microvascular reconstruction was quite challenging [10]. Limited access through such narrow passages made adequate exposure difficult, and conventional methods utilized mandibulotomy or mandibular splitting with considerably high comorbidities, complications, and poor aesthetic outcomes [25].

The rise of trans-oral robotic surgery with the use of the Da Vinci robotic system is an emerging practice in cavity tumour resection, where expertise is available. Although FDA approval restricts its use to tumor sizes T1 and T2 [9], this has continued to yield favorable surgical outcomes.

Microsurgery

Microsurgery robotic systems, for example, the Symani Surgical system, have facilitated complex reconstructions involving multiple microvascular anastomoses. Dastagir et al. reported that the estimated anastomosis surgery length with the Symani (arterial 17.3 ± 1.9 min; venous 11.5 ± 1.3 min) was comparable to the hand-sewn technique (arterial 16.1 ± 1.4 min; venous 10.2 ± 1.8 min) [26].

With these outcomes, there is increasing integration of robotic systems in emergency reconstructive settings, for example, limb salvage following trauma [26].

Struebing et al. in a prospective cohort study reported high safety and reliability with robot-assisted microsurgery and a lower rate of microvascular compromise compared to conventional methods [4]. This is the current direction of modern medicine and portrays an asset in trauma and reconstructive practice (see Table 1).

**Table 1:** Summary of key studies on robotic-assisted surgery in plastic and reconstructive procedures

Study/author (year)	Procedure/focus	Key findings	Reference
Kawashima et al. (2025)	Robotic targeting system in breast surgery	Mean targeting accuracy of 1.158 mm	(27)
Yusufov et al. (2025)	Meta-analysis of DIEP and LD flaps	RAS: longer OR time, shorter hospital stay	(7)
Könneker et al. (2025)	Robotic-assisted extremity reconstructions	High precision in vascular dissection	(16)
Tolksdorf et al. (2024)	Robotic vs. traditional in craniofacial surgery	No difference in flap loss/revision vs traditional	(15)
Pérez-Salazar et al. (2024)	Ergonomic analysis of robotic vs laparoscopy	Improved posture, reduced musculoskeletal stress	(18)
Struebing et al. (2024)	100 cases of robotic microsurgery	2.7% flap loss, 3% required conversion	(20)
Dastagir et al. (2024)	Symani in emergency trauma care	Comparable anastomosis time, safe in trauma	(26)
Awad et al. (2024)	Meta-analysis of RAS vs. conventional in breast reconstruction	Reduced hospital stay, comparable safety	(10)
Innocenti et al. (2023)	First-in-human robotic free flap	Success in 0.8 mm vessels, no re-op needed	(21)
Roy et al. (2023)	Review on robotic flap harvest in breast reconstruction	RAS had fewer complications and higher satisfaction	(11)
Van Mulken et al. (2020)	RCT on Symani robotic supermicrosurgery	Significantly fewer errors, higher-quality anastomosis	(2)

Lymphovenous anastomosis

Robot-assisted super microsurgery enhanced with microscopic visualization offers a superior advantage in lymphovenous anastomosis. Awad et al. described better ease and precision with robot-assisted end-to-end anastomosis [10]. Lymphedema surgeries are conventionally difficult operations due to the minute sutures and instrumentation. Kawashima et al. reported widening indications for this high-level precision RAS [27], enabling interventions that previously were considered impractical.

Breast reconstruction

RAS harvesting of DIEP and LD flaps for breast reconstruction has been associated with a statistically significant reduction in duration of postoperative inpatient stay and reduced postoperative pain [2]. The use of a single axillary access incision accommodating multiple robotic instruments utilized in robotic nipple sparing mastectomy has shown higher patient satisfaction with scar outcomes [8,10], reflecting the current shift toward minimally invasive approaches.

Patient perception, acceptance, and technological innovations on the horizon

Evaluating patient perceptions and experiences with these modern techniques is important. One of the desired outcomes in plastic and reconstructive surgery is the ability to leave as little trace of surgical intervention as possible. While this is dependent on the type of surgery, availability of equipment, postoperative care, and the surgeon’s experience and technique, RAS has improved the feasibility of this outcome. As highlighted in this narrative review, RAS improves access and visualization in difficult areas and filters tremors, enabling more precise surgery and providing better results, shorter recovery time, and more positive patient perceptions. Robotic-assisted approaches demonstrate improved precision, aesthetic outcomes, comparable flap-survival rates, and high patient satisfaction, particularly in procedures involving lymphaticovenous anastomosis and nipple-sparing mastectomy [12].

As mentioned, the use of robotics in breast surgical procedures has been one of the fields of greatest growth in terms of the use of robotics, specifically in plastic and reconstructive surgery, mostly because robotic techniques offer significant benefits in reconstructive breast surgery, allowing surgeons to operate through smaller incisions, resulting in reduced scarring and more aesthetically pleasing results [28]. The final outcomes include reduced scarring, decreased pain, and accelerated patient recovery time [29], thereby promoting greater patient acceptance of surgery performed with modern techniques.

The degree of patient acceptance and understanding of robotic surgery is also linked to communication between medical teams and patients. A 2022 opinion survey among plastic surgeons revealed that the majority held favorable views of robotic surgery and believed in its potential to improve surgical outcomes [30]. Surgeons’ opinions of robotic techniques have become progressively more favorable; this trend, in turn, has fostered patient trust and improved acceptance of robotics.

RAS in plastic and reconstructive surgery continues to emerge, and with the use of mixed-reality innovations, for instance, digital data can be manipulated in vivo to plan thin anterolateral thigh perforator free flaps with a 100% success rate [31]. Some of the disadvantages of robotic surgical equipment, such as its large size, are being addressed through ongoing technological improvements, creating more compact and versatile systems, making them more accessible for a wider range of surgical procedures. However, there is still the existing limitation of cost and regulatory barriers.

Enhanced training platforms, such as virtual reality (VR) simulators and other robotic training platforms, are widely used to train surgeons in robotic techniques; these VR platforms are expected to become more sophisticated, allowing surgeons to acquire and refine robotic skills more effectively, ultimately improving patient outcomes and reducing hospital stays.

Challenges and limitations of the study

Despite the robust potential benefits of RAS in plastic and reconstructive surgery, it remains an evolving field and faces certain challenges and limitations.

The majority of the literature consists of case reports, small-scale studies, or feasibility studies. Furthermore, available literature also demonstrates non-uniformity in the patient population, procedures, outcome measures, and lengths of follow-up, posing challenges to drawing definitive comparative conclusions on the efficacy of robotic versus traditional methods [4].

Another limitation of the literature is the risk of publication bias, which is not uncommon in an emerging field aiming to establish clinical relevance and feasibility. Negative or inconclusive reports may be under-reported.

Another aspect is the integration of robotics in plastic surgery, which brings its own set of limitations. The absence of tactile feedback is a widely acknowledged limitation of existing robotic systems. Microsurgery demands delicate tissue handling and suture tension, which tactile feedback provides. In the absence of this sensory feedback, operating surgeons must rely on visual cues, posing challenges in complex tissue manipulations [4,10,12,27].

There is also a lack of instrumentation designed specifically for microsurgery. Magnifications of the optical system of the robotic systems still need improvement compared to conventional optical microscopes, particularly in super microsurgery, lymphatic surgery, and complex neural repair, where fine suture handling, delicate tissue manipulation, and ultra-high magnification are essential. Although some newer systems are emerging, including robotic platforms purpose-built for microsurgery, widespread clinical experience with these tools is still limited [9].

Several studies report longer operating time compared to conventional techniques, largely due to system setup times and steep early-stage learning curves [2,12].

**Figure 2:**
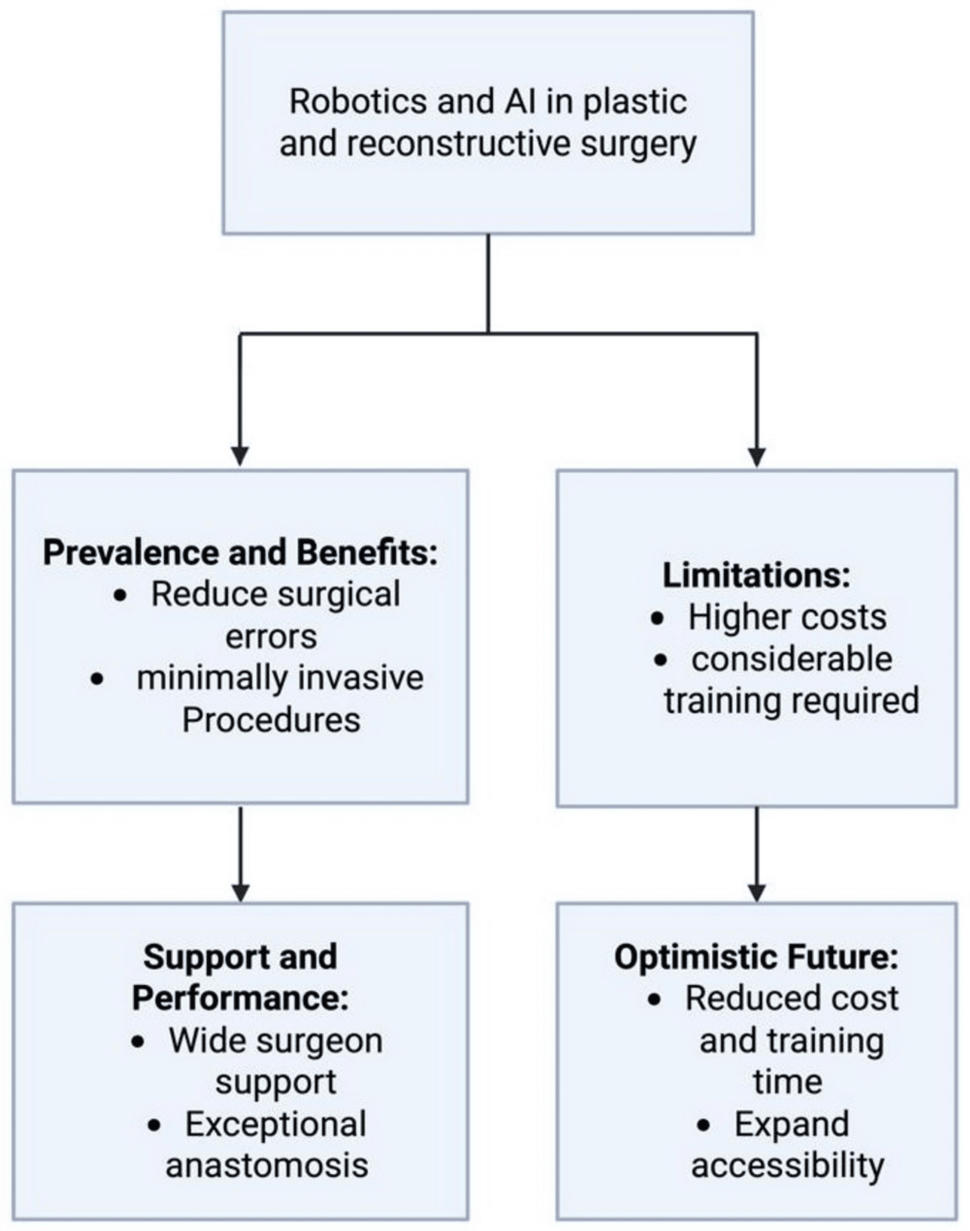
Benefits and limitations of robotic-assisted surgery in plastic and reconstructive surgery Created in BioRender. Alkarboli Y (2025). https://www.biorender.com Publication License ID: WV28VH6OM2

Cost is another major limitation. The financial demands of purchasing, maintaining, and operating robotic platforms are substantial. Moreover, consumables and ongoing training for both surgeons and operating teams add to these costs. Currently, many plastic surgery departments lack financial resources to integrate robotic systems into everyday practice [12], particularly in environments where healthcare budgets are tightly constrained and reimbursement for robotic surgery is uncertain (Figure 2).

Practical considerations such as the substantial space requirements of robotic systems and their integration into existing operating theatre workflows present further hurdles [10]. Many operating rooms optimized for conventional microsurgery lack the capacity or infrastructure to accommodate large robotic platforms without significant redesign [12,32].

Finally, ethical challenges exist. Core issues such as informed consent for robotics-driven procedures, data protection, and privacy need to be addressed. Furthermore, a clear delineation of liability and accountability should adverse events occur is needed. There is also the possibility of robot malfunction, software errors, or system hacking, further complicating the ethical landscape [9,33,34].

How will robotics impact the future of plastic and reconstructive surgery?

The transformative potential of technology in robotic surgery will shape the future of plastic and reconstructive surgery, for excellent patient care and to improve the efficiency of the surgical team. There is a positive impact on the multidisciplinary team, including surgeons, nurses, technicians, anesthesiologists, and support staff, improving efficiency and decreasing stress [16,29].

RAS demonstrates significantly higher patient satisfaction owing to minimal scarring and advanced precision. Another advantage includes the use of telemanipulation capabilities of robotic surgery to facilitate remote procedures, increasing access to specialized care [35]. This technology will be beneficial where strict social limitations are in place to limit infectious disease spread - a benefit highlighted during the COVID-19 pandemic, when elective procedures were deferred to limit infection spread. In future health crises, telepresence may mitigate similar constraints [29].

Although traditional techniques are still dominant in plastic and reconstructive surgery, a recent study shows that 89.7% of surgeons support using robots intra-operatively, with 56.4% being used in pelvic/perineal reconstruction, 43.6% in abdominal reconstruction, and 44.2% in super-microsurgery [29]. Another study highlights the outstanding safety and efficacy of the Symani system in micro-anastomosis, proving it would be a better option for complex reconstructive procedures [2,16].

As the technology continues to advance, more robotic adjuvant tools are emerging. Proposed enhancements include optical magnification and projection systems, virtual reality systems, NIRF (near-infrared technology), imaging technologies, and concomitant use of confocal microscopy that can create imaging at a near cellular level [9]. Other more advanced tools include micro-Doppler probes with artery and vein mapping, robotic hydrojet devices, laser ablation, and biofeedback sensors. The impact of the application of these futuristic adjuvant tools is promising by facilitating real-time intraoperative anatomical navigation and improving outcomes [9].

In addition, integration of artificial intelligence and visual learning in robotic surgery offers the potential for automated tissue recognition, enhanced surgical planning, and intraoperative decision support [22].

Finally, RAS remains an evolving technique in plastic surgery; the increasing amount of research indicates that it is not merely a prospective choice but a present and expanding solution.

## Conclusions

Plastic and reconstructive surgery is undergoing a transformative change through RAS, which provides surgeons with precise control, better ergonomic conditions, and better access to complex anatomical areas. RAS demonstrates its safety and effectiveness in reconstructive procedures, including breast reconstruction and microsurgical trauma repair, which produce results equivalent to or better than conventional surgical techniques. The implementation of RAS results in decreased postoperative complications and shorter hospital stays alongside better aesthetic results and better patient satisfaction rates. However, the adoption of RAS faces multiple barriers that prevent its widespread use. High financial costs, lack of tactile feedback, steep learning curves, and limited instrumentation for super microsurgery constrain its widespread adoption.

The current literature base is fragmented with mostly small-scale studies and heterogeneous methodologies. Standardized high-quality comparative trials must be conducted to prove the clinical value of RAS across different subspecialties in plastics surgery. The development of virtual reality, artificial intelligence, and miniaturized robotic systems will enhance both accessibility and cost-effectiveness of RAS. For the complete realization of this technology, strategic funding for surgeon training initiatives, infrastructure development, alongside strong clinical research programs is essential. With appropriate integration and refinement, RAS will become an essential surgical method in modern plastic and reconstructive surgery through its combination of precise surgical techniques with safe practices and patient-oriented care.
